# Cryptolepine Suppresses Colorectal Cancer Cell Proliferation, Stemness, and Metastatic Processes by Inhibiting WNT/β-Catenin Signaling

**DOI:** 10.3390/ph16071026

**Published:** 2023-07-19

**Authors:** Jude Tetteh Quarshie, Kwadwo Fosu, Nicholas Awuku Offei, Augustine Kojo Sobo, Osbourne Quaye, Anastasia Rosebud Aikins

**Affiliations:** West African Centre for Cell Biology of Infectious Pathogens (WACCBIP), Department of Biochemistry Cell and Molecular Biology, University of Ghana, Accra P.O. Box LG 54, Ghana

**Keywords:** cryptolepine, WNT/β-catenin signaling, colorectal cancer, proliferation, stemness, metastasis

## Abstract

Colorectal cancer (CRC) is the third most frequent cancer and the second leading cause of cancer-related deaths globally. Evidence shows that over 90% of CRC cases are initiated by a deregulated Wingless Integrated Type-1 (WNT)/β-catenin signaling pathway. The WNT/β-catenin pathway also promotes CRC cell proliferation, stemness, and metastasis. Therefore, modulators of the WNT/β-catenin pathway may serve as promising regimens for CRC. This study investigated the effect of cryptolepine—a plant-derived compound—on the WNT/β-catenin pathway in CRC. Two CRC cell lines, COLO205 and DLD1, were treated with cryptolepine or XAV 939 (a WNT inhibitor) in the presence or absence of WNT3a (a WNT activator). Using a tetrazolium-based assay, cryptolepine was found to reduce cell viability in a dose- and time-dependent manner and was a more potent inhibitor of viability than XAV 939. RT-qPCR analyses showed that cryptolepine reverses WNT3a-induced expression of *β-catenin*, *c-MYC*, and *WISP1*, suggesting that cryptolepine inhibits WNT3a-mediated activation of WNT/β-catenin signaling. Cryptolepine also repressed WNT3a-induced *OCT4* and *CD133* expression and suppressed colony formation of the cells, indicating that cryptolepine inhibits the stemness of CRC cells. Additionally, cryptolepine inhibited WNT3a-induced epithelial-to-mesenchymal transition by reducing the expression of *SNAI1* and *TWIST1* genes. In a wound healing assay, cryptolepine was found to suppress cell migration under unstimulated and WNT3a-stimulated conditions. Moreover, cryptolepine downregulated WNT3a-induced expression of *MMP2* and *MMP9* genes, which are involved in cancer cell invasion. Altogether, cryptolepine suppresses CRC cell proliferation, stemness, and metastatic properties by inhibiting WNT3a-mediated activation of the WNT/β-catenin signaling pathway. These findings provide a rationale for considering cryptolepine as a potential WNT inhibitor in CRC.

## 1. Introduction

In the year 2020, colorectal cancer (CRC) accounted for 1.9 million new cancer cases and 935,000 cancer-related deaths [1]. It causes severe morbidity in affected persons, and the probability of survival depends on the stage at diagnosis [2]. Research is ongoing to find the etiology of CRC, and although this remains elusive, evidence shows that CRC is initiated when genetic and epigenetic alterations in oncogenes and tumor suppressor genes accumulate in mucosal epithelial cells of the intestines [3]. Predisposing factors include gender (males are more susceptible than females), obesity, a sedentary lifestyle, consumption of high amounts of alcohol and red meat, and smoking cigarettes. Protective factors include the consumption of whole grains, dairy products, and fibers [4].

In the healthy human gut, the entire lining of the colonic lumen self-renews weekly to replace the glandular epithelial cells that are lost to stress from digestion and evacuation. This is made possible by the abundant intestinal stem cells at the bottom of the intestinal crypts, which have the capacity for continual proliferation and differentiation. As these pluripotent cells differentiate into epithelial cells, they migrate from the crypt to the villus and become fully differentiated epithelial cells such as enterocytes, goblet cells, Paneth cells, and enteroendocrine cells [5]. In the event of oncogenic insults, the epithelial cells lose their ability to properly regulate proliferation and transform into a benign adenoma that may turn cancerous (adenocarcinoma) and metastasize over time [2].

Among the suite of signaling cascades that drive CRC, the canonical Wingless Integrated Type-1 (WNT) pathway is principal. Indeed, over 90% of CRC cases occur due to driver mutations that result in constitutive activation of the WNT signaling pathway. The pathway is an evolutionarily conserved pathway that can either be transduced via canonical (WNT/β-catenin) or non-canonical cascades. It is activated when WNT proteins—a family of secreted cysteine-rich glycoproteins—activate their cell surface receptors, known as Frizzled (FZD) [6,7]. In the canonical WNT cascade, WNT ligands bind FZD, which in turn propagates a signal via Dishevelled to cause cytosolic accumulation of β-catenin. Accumulated β-catenin translocates into the nucleus where it forms heterodimers with T cell factor/lymphoid enhancer factor (TCF/LEF) to upregulate the transcription of genes such as *Axin2*, *c-MYC*, *cyclin D1*, *WISP1*, and *OCT4*. These genes promote the proliferation, survival, apoptosis, and stemness of the CRC cells [7,8]. Constitutive activation of WNT signaling confers a clonal advantage to CRC cells, which increases their fitness through modification of the tumor microenvironment [9]. Additionally, WNT/β-catenin signaling promotes stem cell-like properties that are associated with drug resistance and tumor recurrence [5]. Considering the significance of WNT/β-catenin signaling in CRC development and progression, WNT-modulating compounds may serve as promising regimens for CRC.

Due to their chemical diversity, some studies are focusing on naturally occurring compounds in plants and their structural analogs as sources of pharmaceutically useful small organic molecules for cancer treatment. One such compound is cryptolepine, the main alkaloid extracted from the roots of an African-indigenous shrub, *Cryptolepis sanguinolenta* [10,11]. Cryptolepine is pharmacologically active and a potential antiplasmodial, antibacterial, antiprotozoal, antifungal, anti-hyperglycemic, antithrombotic, anti-inflammatory, and anticancer agent [10,12]. The anticancer property of cryptolepine is particularly of interest because, unlike many anticancer agents, cryptolepine exhibits low mutagenic and genotoxic effects [13]. Moreover, a study showed that cryptolepine is highly selective in inhibiting the viability of skin cancer cells versus normal cells [14]. Another study reported no abnormalities in the hematological, liver, and kidney function indices within 6 months of cryptolepine exposure in a 49-year-old male [15], suggesting that it may be a safe alternative for cancer treatment.

Although the molecular basis for its anticancer activity remains largely ambiguous, mechanistic studies have shown that cryptolepine acts through mechanisms such as topoisomerase II and telomerase inhibition, as well as DNA intercalation [16,17,18]. Using hepatocellular carcinoma cell models, Domfeh et al. (2021) demonstrated that cryptolepine mediates its anticancer activity by upregulating anticancer pathway genes and downregulating pro-cancer pathway genes. In their study, it was found that cryptolepine inhibits the transcriptional activity of the TCF/LEF transcription factor [19]. Given the modulatory activity of cryptolepine on WNT/β-catenin signaling, it is imperative to assess its effect on CRC models. In this study, we demonstrate that cryptolepine suppresses CRC cell proliferation, stemness, and metastatic processes through inhibition of the WNT/β-catenin signaling pathway.

## 2. Results

### 2.1. Effects of Cryptolepine, XAV 939, and WNT3a on Cell Viability

**Figure 1:**
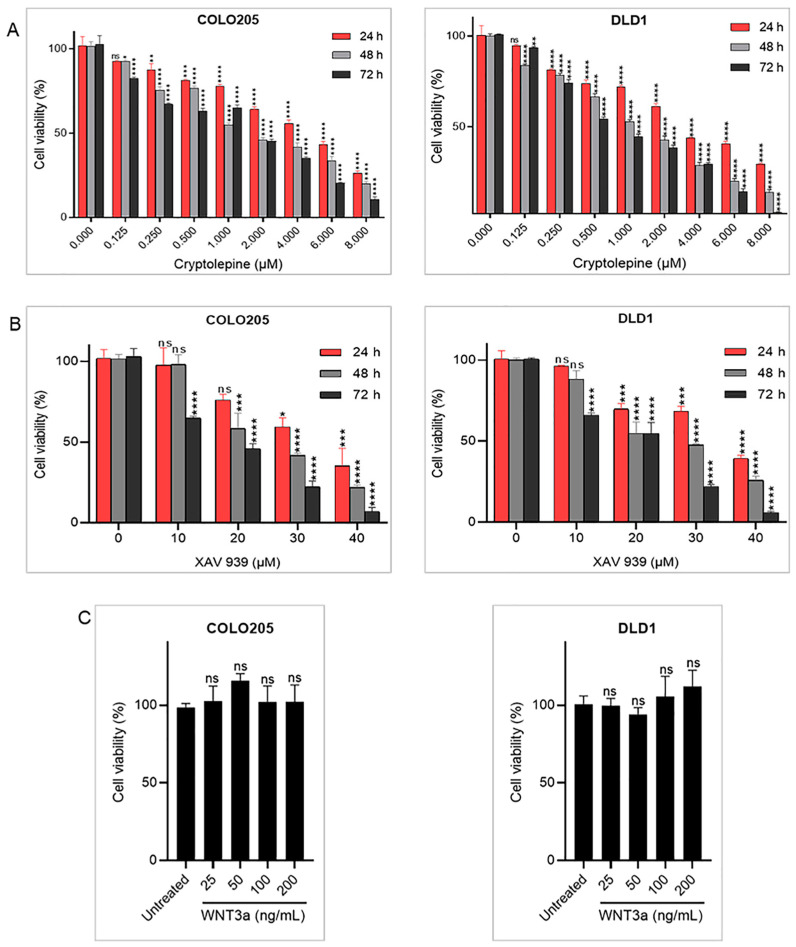
Effects of cryptolepine, XAV 939, and WNT3a on cell viability. COLO205 and DLD1 cells were treated with increasing concentrations of (**A**) cryptolepine or (**B**) XAV 939 for 24, 48, and 72 h, or (**C**) WNT3a for 24 h. Cell viability was determined using an MTT assay. Data are presented as mean ± SEM of three independent experiments performed in triplicate. * *p* ≤ 0.05, ** *p* ≤ 0.01, *** *p* ≤ 0.001, **** *p* ≤ 0.0001 vs. untreated. ns: not significant.

To determine the effects of cryptolepine, XAV 939, and WNT3a on cell viability, an MTT assay was performed. Cryptolepine and XAV 939 reduced cell viability in a dose- and time-dependent manner. The IC50 values shown in Table 1 indicate that cryptolepine is more cytotoxic than XAV 939. Furthermore, cryptolepine was more cytotoxic against DLD1 than COLO205 cells, whereas the potency of XAV 939 was identical in both cell lines (Figure 1A,B). WNT3a had no obvious effect on the viability of both cell lines (Figure 1C). Based on a literature search [20,21,22], 100 ng/mL of WNT3a was used to activate WNT signaling in further experiments.

### 2.2. Cryptolepine Inhibits WNT/β-Catenin Signaling in CRC Cells

**Figure 2:**
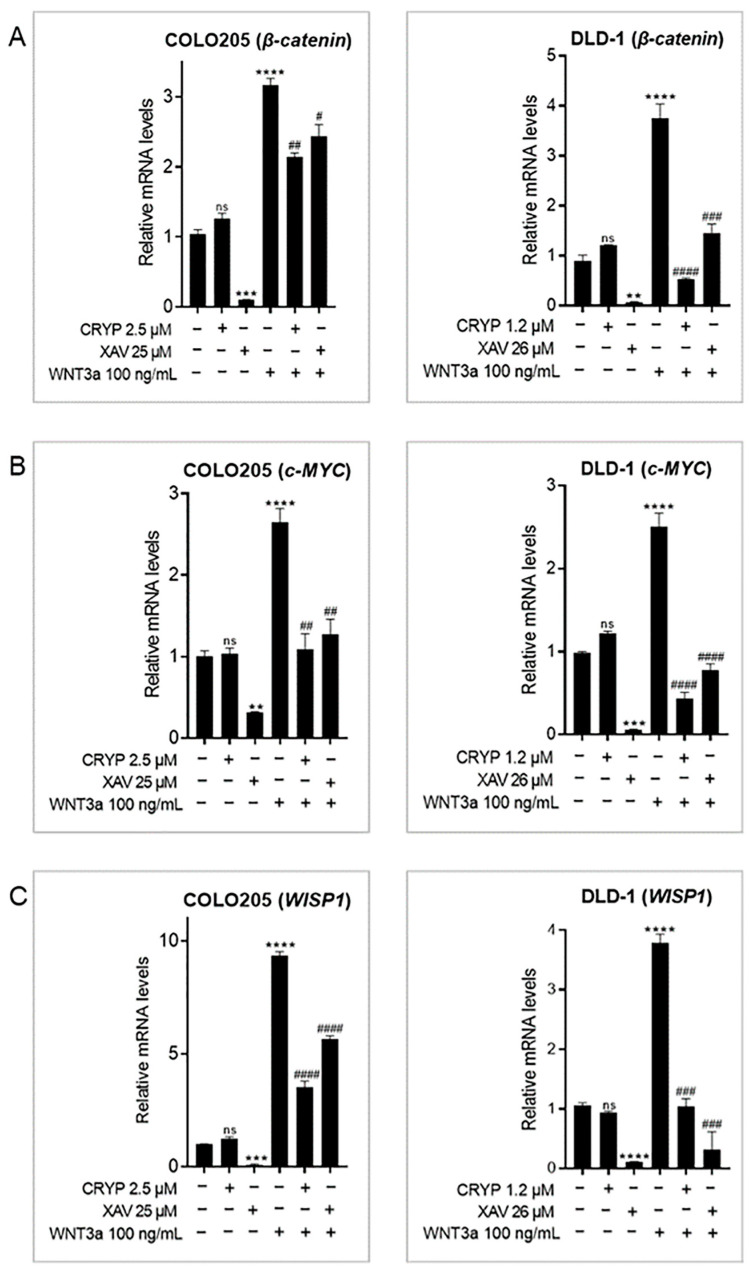
Cryptolepine inhibits WNT/β-catenin signaling in CRC cells. COLO205 and DLD1 cells were treated with cryptolepine or XAV 939 for 48 h in the presence or absence of WNT3a. The mRNA levels of (**A**) *β-catenin*, (**B**) *c-MYC*, and (**C**) *WISP1* were determined by RT-qPCR. Data are presented as mean ± SEM of three independent experiments performed in triplicate. ** *p* ≤ 0.01, *** *p* ≤ 0.001, **** *p* ≤ 0.0001 vs. untreated. # *p* ≤ 0.05, ## *p* ≤ 0.01, ### *p* ≤ 0.001, #### *p* ≤ 0.0001 vs. WNT3a. CRYP: cryptolepine; XAV: XAV 939; mRNA: messenger ribonucleic acid; ns: not significant.

To assess the effect of cryptolepine on WNT/β-catenin signaling, the cells were treated as described, and mRNA levels of *β-catenin*, *c-MYC*, and *WISP1* were evaluated [7]. WNT3a induced the expression of *β-catenin*, *c-MYC*, and *WISP1*, while XAV 939 inhibited their expression. Cryptolepine did not affect the basal levels of all three genes in both cell lines but significantly suppressed WNT3a-induced expression of the genes (Figure 2A–C). This suggests that cryptolepine inhibits WNT3a-mediated activation of the WNT/β-catenin pathway.

### 2.3. Cryptolepine Downregulates Stem Cell Markers in CRC

**Figure 3:**
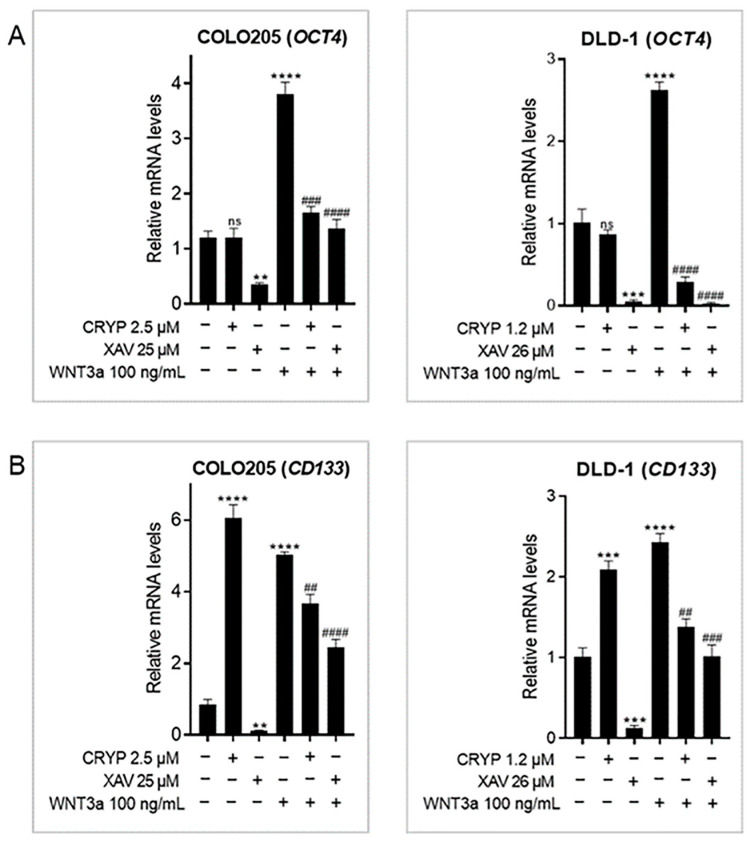
Cryptolepine downregulates stem cell markers in CRC. COLO205 and DLD1 cells were treated with cryptolepine or XAV 939 for 48 h in the presence or absence of WNT3a. The mRNA levels of (**A**) *OCT4* and (**B**) *CD133* were determined by RT-qPCR. Data are presented as mean ± SEM of three independent experiments performed in triplicate. ** *p* ≤ 0.01, *** *p* ≤ 0.001, **** *p* ≤ 0.0001 vs. untreated. ## *p* ≤ 0.01, ### *p* ≤ 0.001, #### *p* ≤ 0.0001 vs. WNT3a. CRYP: cryptolepine; XAV: XAV 939; mRNA: messenger ribonucleic acid; ns: not significant.

Aberrant WNT signals promote stemness in CRC, which is linked to poor disease outcomes [23]. To this end, the impact of cryptolepine on the expression of two stem cell markers—*OCT4* and *CD133*—was examined [7]. WNT3a induced the expression of *OCT4* and *CD133* in both cell lines and XAV 939 decreased their expression in unstimulated and WNT3a-stimulated cells (Figure 3A,B). Cryptolepine did not affect the basal level of *OCT4* in both cells but suppressed WNT3a-induced *OCT4* expression (Figure 3A). Interestingly, cryptolepine induced *CD133* expression in both cells but repressed WNT3a-induced *CD133* expression (Figure 3B), suggesting that cryptolepine may promote or suppress *CD133* expression depending on WNT activity in the cell.

### 2.4. Cryptolepine Inhibits Clonogenicity in CRC

**Figure 4:**
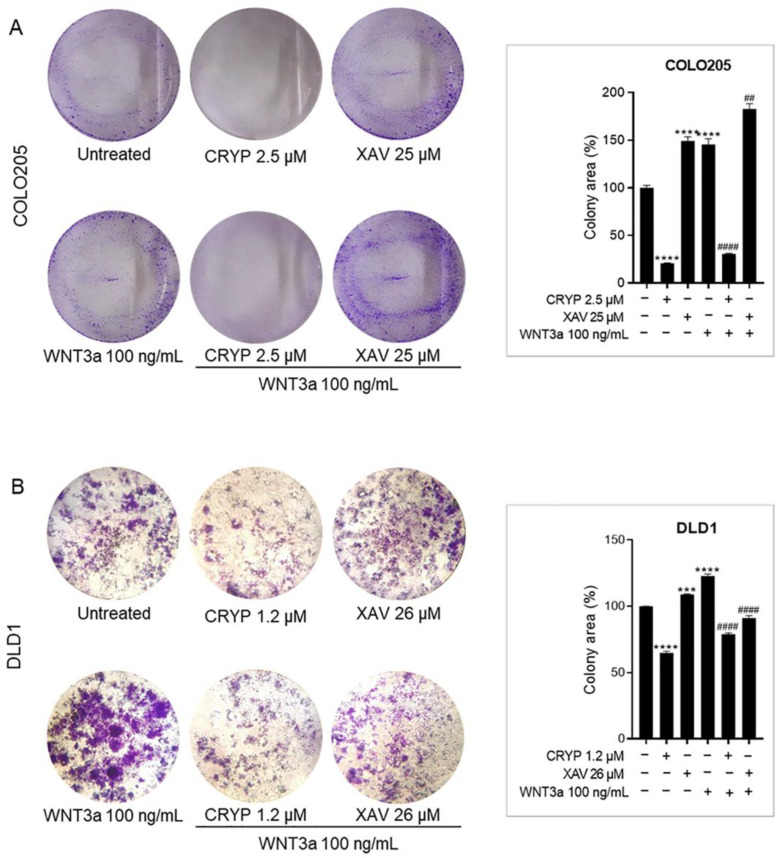
Cryptolepine inhibits clonogenicity in CRC. (**A**) COLO205 and (**B**) DLD1 cells were treated with cryptolepine or XAV 939 in the presence or absence of WNT3a, and a colony formation assay was performed. Colony areas are expressed as a percentage of untreated cells and presented as bar charts. Data are presented as mean ± SEM of three independent experiments performed in triplicate. *** *p* ≤ 0.001, **** *p* ≤ 0.0001 vs. untreated. ## *p* ≤ 0.01, #### *p* ≤ 0.0001 vs. WNT3a. CRYP: cryptolepine; XAV: XAV 939.

Given the inhibitory effect of cryptolepine on the expression of stemness markers, we assessed its influence on the colony-forming ability of CRC cells. The assay assesses the capacity of a single cell to grow into a colony of at least 50 cells, which signifies stemness based on the rationale that following treatment, stem cells usually persist and regenerate to initiate tumorigenesis [24]. In this study, WNT3a promoted colony formation in both cell lines, confirming the influence of WNT signaling on stemness in CRC cells. Surprisingly, XAV 939 increased the colony formation of unstimulated cells and repressed WNT3a-induced clonogenicity in DLD1 cells but not COLO205 cells. On the other hand, cryptolepine suppressed clonogenicity in both unstimulated and WNT3a-stimulated COLO205 and DLD1 cells, suggesting that cryptolepine inhibits colony formation and hence stem cell-like properties of the CRC cells (Figure 4A,B).

### 2.5. Cryptolepine Represses Epithelial-to-Mesenchymal Transition (EMT) in CRC

**Figure 5:**
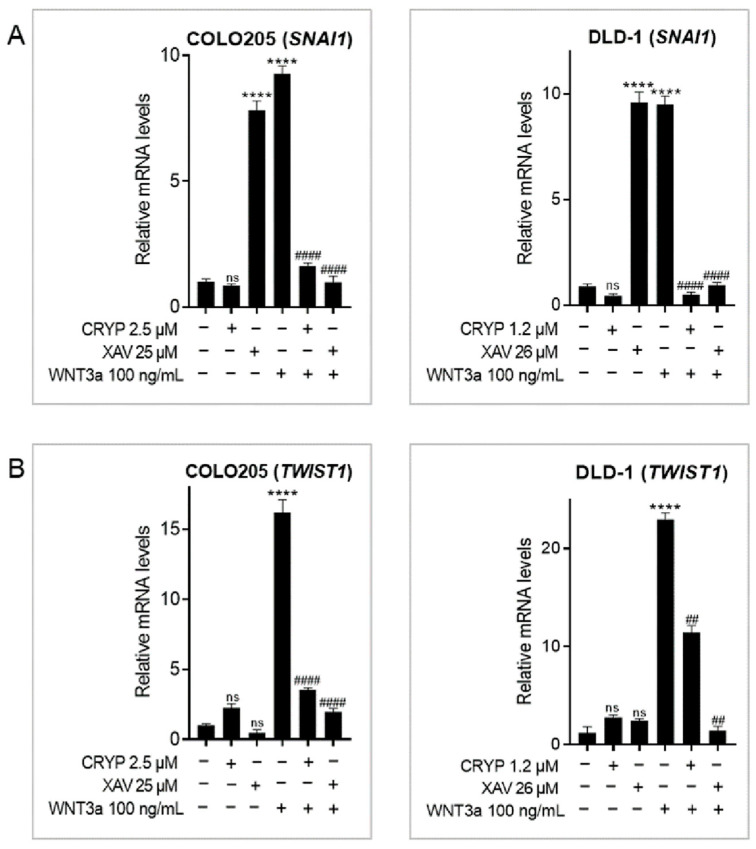
Cryptolepine represses epithelial-to-mesenchymal transition (EMT) in CRC. COLO205 and DLD1 cells were treated with cryptolepine or XAV 939 for 48 h in the presence or absence of WNT3a. The mRNA levels of (**A**) *SNAI1* and (**B**) *TWIST1* were determined by RT-qPCR. Data are presented as mean ± SEM of three independent experiments performed in triplicate. **** *p* ≤ 0.0001 vs. untreated. ## *p* ≤ 0.01, #### *p* ≤ 0.0001 vs. WNT3a. CRYP: cryptolepine; XAV: XAV 939; mRNA: messenger ribonucleic acid; ns: not significant.

Metastasis is a multistep process that involves landmark events like EMT, where cancer cells become highly motile. Because deregulated WNT/β-catenin signaling drives EMT in CRC [25], the expression of two EMT genes—*SNAI1* (encodes SNAIL1 protein) and *TWIST1*—were examined. WNT3a induced the expression of SNAI1 and TWIST1 genes in both cell lines. XAV 939 induced the expression of *SNAI1* in both cell lines, whereas cryptolepine did not affect *SNAI1* levels in unstimulated cells. In contrast, both XAV 939 and cryptolepine significantly inhibited WNT3a-induced *SNAI1* expression (Figure 5A). Furthermore, XAV 939 and cryptolepine did not affect the basal levels of *TWIST1* mRNA in unstimulated cells but suppressed WNT3a-induced *TWIST1* upregulation (Figure 5B). The results, therefore, show that cryptolepine represses EMT in CRC cells with hyperactive WNT signaling via downregulation of *SNAI1* and *TWIST1*.

### 2.6. Cryptolepine Reduces CRC Cell Migration

**Figure 6:**
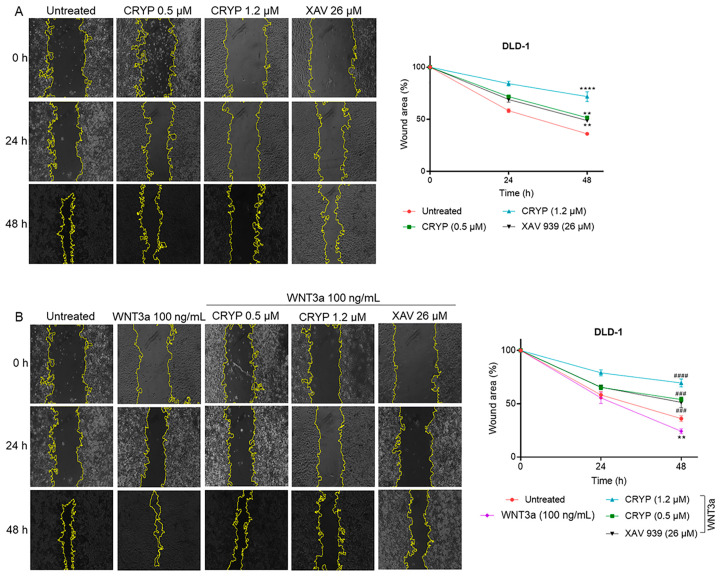
Cryptolepine reduces CRC cell migration. DLD1 cells were treated with cryptolepine or XAV 939 for 48 h in the (**A**) presence or (**B**) absence of WNT3a. The wound area at each time point is expressed as a percentage of the 0 h. Original magnification ×100. Data are presented as mean ± SEM of three independent experiments performed in triplicate. ** *p* ≤ 0.01, **** *p* ≤ 0.0001 vs. untreated. ### *p* ≤ 0.001, #### *p* ≤ 0.0001 vs. WNT3a. CRYP: cryptolepine; XAV: XAV 939.

A wound healing assay was performed to evaluate the effect of cryptolepine on cell migration. DLD1 cells were treated with IC50 and IC30 of cryptolepine or IC50 of XAV 939 in the presence or absence of WNT3a. Cell migration was monitored at 0, 24, and 48 h. Activating or inhibiting WNT/β-catenin signaling promoted or inhibited cell migration, respectively. The IC30 and IC50 of cryptolepine remarkably inhibited wound closure in both unstimulated and WNT3a-stimulated DLD1 cells (Figure 6A,B). These findings demonstrate that cryptolepine is a potent inhibitor of DLD1 cell migration.

### 2.7. Cryptolepine Reduces CRC Cell Invasiveness

**Figure 7:**
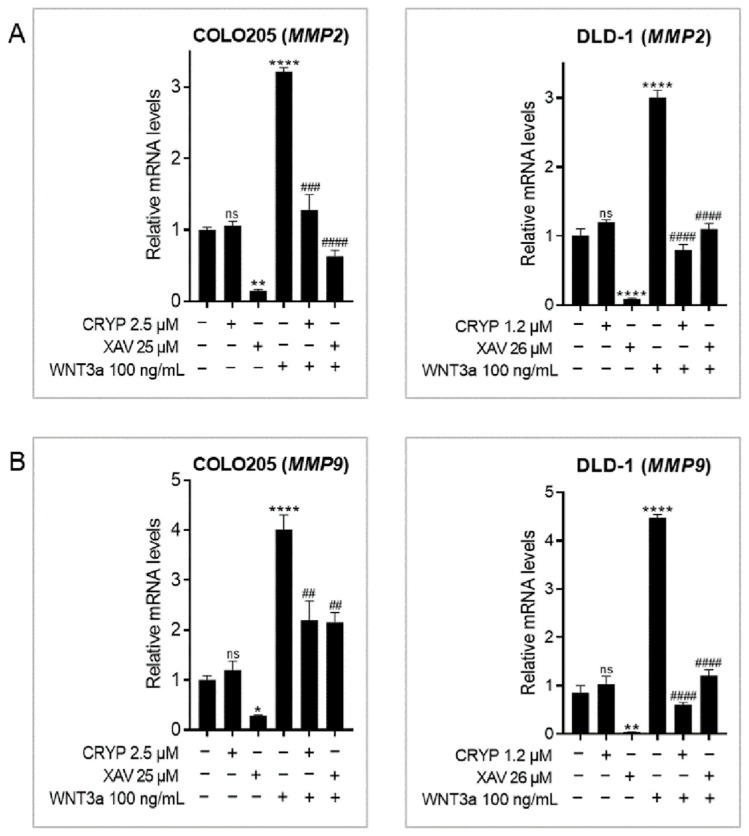
Cryptolepine reduces CRC cell invasiveness. COLO205 and DLD1 cells were treated with cryptolepine or XAV 939 for 48 h in the presence or absence of WNT3a. The mRNA levels of (**A**) *MMP2* and (**B**) *MMP9* were determined by RT-qPCR. Data are presented as mean ± SEM of three independent experiments performed in triplicate. * *p* ≤ 0.05, ** *p* ≤ 0.01, **** *p* ≤ 0.0001 vs. untreated. ## *p* ≤ 0.01, ### *p* ≤ 0.001, #### *p* ≤ 0.0001 vs. WNT3a. CRYP: cryptolepine; XAV: XAV 939; mRNA: messenger ribonucleic acid; ns: not significant.

To examine the impact of cryptolepine on cell invasion, the levels of *MMP2* and *MMP9* were determined. These genes encode proteinases that degrade the histological barrier to facilitate cancer cell invasion [26]. Activating or inhibiting WNT/β-catenin signaling led to an upregulation or downregulation, respectively, of *MMP2* and *MMP9* in both cell lines. Cryptolepine had no obvious effects on *MMP2* and *MMP9* expression in unstimulated cells but significantly reduced WNT3a-induced expression of these genes, confirming the selectivity of cryptolepine for cancer cells with hyperactive WNT signaling (Figure 7A,B).

## 3. Discussion

The importance of WNT/β-catenin signaling in CRC has been established [9], and research is still ongoing to develop/discover small molecule inhibitors of the pathway to treat CRC. Cryptolepine is a plant-derived compound that has been found to inhibit the transcriptional activity of TCF/LEF protein (a transcriptional factor in WNT/β-catenin signaling) in hepatocellular carcinoma cells [19]. This study, therefore, investigated the potential of cryptolepine as a modulator of WNT/β-catenin signaling in CRC cells.

In this study, cryptolepine exhibited more potent cytotoxic effects than XAV 939. Cryptolepine targets DNA, topoisomerase II, and telomerase, and affects a myriad of signaling pathways [16,17,18,19]. XAV 939, on the other hand, is a tankyrase inhibitor that specifically inhibits WNT signaling [27]. The superior cytotoxic effect of cryptolepine may therefore be attributed to its ability to regulate multiple signaling pathways simultaneously. Surprisingly, stimulating WNT activity in the CRC cells with recombinant WNT3a had no obvious effect on viability. In their study, Reischmann et al., (2015) found that, although WNT3a enhances HEK293 cell proliferation, this effect can only be detected by measuring DNA synthesis (BrdU assay) rather than mitochondrial activity (MTT assay) [28]. While this study did not assess the proliferative effects of WNT3a using other assays, the apparent ineffectiveness of WNT3a may be ascribed to limitations in the method of measuring cell viability i.e., MTT assay.

β-catenin is the main effector in canonical WNT signaling. It interacts with TCF/LEF and other proteins to induce the transcription of WNT-target genes [29]. The effect of tankyrase inhibition on the expression of β-catenin shows that XAV 939 does not only facilitate the degradation of β-catenin protein but also reduces its mRNA level as previously reported [30]—either by inhibiting transcription of the *CTNNB1* gene (encodes for β-catenin) or by facilitating the degradation of *β-catenin* mRNA. Although cryptolepine is reported to suppress the transcriptional activity of TCF/LEF [19], it is not known if cryptolepine indeed affects the WNT/β-catenin signaling pathway, or how it affects the pathway. This study revealed that cryptolepine inhibits WNT3a-induced expression of *β-catenin*, *c-MYC*, and *WISP1*. This may explain the findings by Domfeh et al. (2021) who showed that cryptolepine suppresses the transcriptional activity of both TCF/LEF and MYC proteins [19]. Additionally, our findings are consistent with previous reports in which cryptolepine reduced c-MYC levels in melanoma cells [31]. In CRC, deregulated c-MYC is indispensable in cell growth and proliferation and is linked to unfavorable prognosis and poor patient survival [32]. WISP1 also enhances survival by protecting cancer cells from p53-mediated apoptosis [33]. The inhibitory effect of cryptolepine on the expression of these genes is therefore favorable for CRC treatment. Moreover, cryptolepine suppressed WNT/β-catenin signaling in WNT3a-stimulated cells only. We, therefore, hypothesize that the influence of cryptolepine on the WNT/β-catenin pathway is selective for cells with hyperactive WNT signals.

Colorectal cancer stem cells (CSCs) express cell surface markers such as OCT4 and CD133 (AC133 or prominin 1). OCT4 promotes stem cell maintenance and immune evasion and positively correlates with liver metastasis in CRC [34,35]. CRC cells expressing CD133 are more resistant to radiochemotherapy and are an indication of worse overall survival and metastatic relapse [36,37]. WNT3a and XAV 939 had opposing effects on the expression of *OCT*4 and *CD133*, supporting evidence on the influence of WNT signaling on the expression of these genes and hence stemness of CRC cells. Cryptolepine reduced the mRNA levels of *OCT4* in WNT3a-stimulated cells, signifying its effect on WNT-meditated OCT4 expression. Treatment of the cells with cryptolepine upregulated basal *CD133* mRNA levels. We propose two theories to explain this observation. First, cryptolepine enhances the transcriptional activity of SMAD2/SMAD3/SMAD4 proteins [19], which drive transforming growth factor β (TGFβ) signaling. TGFβ has been shown to enhance CD133 expression through the demethylation of *CD133* promoter-1 [38]. Thus, cryptolepine increased the expression of *CD133* through the activation of TGFβ signaling. Second, cryptolepine promoted MAPK/ERK signaling by enhancing the transcriptional activity of the serum response element (SRE) protein [19]. Hyper-activated MAPK pathway leads to overexpression of CD133 mRNA and protein in CRC [39]. Therefore, cryptolepine increased *CD133* expression by upregulating MAPK signaling. Experimental evidence is required to validate these theories, especially because cryptolepine also enhances the transcriptional activity of the p53 protein [19], which has been shown to suppress the activity of the CD133 promoter and downregulate CD133 expression [40]. This study also found that cryptolepine reduced WNT3a-induced *CD133* expression, suggesting that it may promote or suppress *CD133* expression depending on WNT activity in the cell.

The current study found that WNT3a induced colony formation in both cell lines. Contrary to previous findings [41,42], tankyrase inhibition increased the colony formation of the cells. This may be attributed to an inability of XAV 939 to exert a long-term effect on the CRC cells, allowing them to regenerate after treatment. Conversely, cryptolepine significantly suppressed the colony-forming ability of unstimulated and WNT3a-stimulated cells. The superior ability of cryptolepine over XAV 939 to inhibit colony formation may be attributed to the capacity of cryptolepine to exert a long-lasting effect on the cells. Moreover, the ability of cryptolepine to inhibit telomerase activity and cause DNA damage may have accounted for this observation, as these events also suppress colony-forming ability [43,44].

One essential step in metastasis is EMT, a phenomenon where cells undergo rearrangement of the cytoskeleton and switch from a non-motile epithelial state to a motile mesenchymal state. EMT facilitates cell migration and generates and maintains CSCs [45]. Several transcription factors, including SNAIL1 and TWIST1, regulate EMT [25]. In this study, WNT3a induced *SNAI1* expression, corroborating previous reports [46,47]. Tankyrase inhibition with XAV 939 upregulated basal *SNAI1* expression in both cell lines. Contrasting findings have been reported in this regard. While some research found that XAV 939 decreased SNAIL1 expression [48,49], other studies found that XAV 939 increased SNAIL1 expression [50,51]. Indeed, XAV 939 stabilizes and increases the abundance of Axin proteins, which in turn activate SNAIL1 expression [47]. This may explain the increase in *SNAI1* expression following tankyrase inhibition. Cryptolepine significantly reduced *SNAI1* mRNA levels in WNT3a-stimulated but not unstimulated cells, demonstrating the ability of cryptolepine to inhibit EMT through WNT signaling. We also found that WNT3a induced *TWIST1* expression, which is consistent with previous studies that identified TWIST1 as a WNT-inducible transcription factor whose expression can be activated by WNT3a [52,53]. Cryptolepine did not affect basal levels of *TWIST1* but reduced WNT3a-induced TWIST1 levels. In cancer, the expression of TWIST1 is associated with lymphatic vessel invasion, lymph node metastasis, and perineural invasion [25]. Additionally, TIWST1 binds to β-catenin and enhances the transcriptional activity of the β-catenin/TCF4 complex [54]. Therefore, the ability of cryptolepine to suppress WNT3a-induced *TWIST1* expression is a favorable outcome.

Cancer cell migration is critical for distant metastasis [45]. The WNT/β-catenin pathway is essential for this phenomenon, hence the impact of cryptolepine on CRC cell migration was studied. Stimulating WNT signaling promoted cell migration, whereas inhibiting WNT signaling suppressed migration. This is supported by reports that WNT signaling supports cell migration, and inhibition of tankyrase activity attenuates WNT3a-induced cell migration [30,55]. Furthermore, cryptolepine remarkably impeded cell migration, both under unstimulated and WNT3a-stimulated conditions. These findings, therefore, indicate that cryptolepine suppresses the migration of CRC cells.

A key event in cancer invasion is the breakdown and remodeling of the extracellular matrix by matrix metalloproteinases (MMPs). In CRC, MMP2 and MMP9 degrade the histological barrier that would normally prevent invasion. Their overexpression is associated with the transition from colon adenoma to adenocarcinoma and is correlated with poor prognosis in CRC [26,56]. In this study, WNT3a induced *MMP2* and *MMP9* expression. Conversely, tankyrase inhibition suppressed the expression of these genes. This corroborates findings that WNT3a upregulates downstream WNT targets MMP2 and MMP9 [53,57], while XAV 939 suppresses their expression [48,49]. Additionally in this study, cryptolepine had no effect on *MMP2* and *MMP9* basal mRNA levels but suppressed WNT3a-induced expression of these genes, supporting the theory that the effect of cryptolepine on the WNT/β-catenin pathway is selective for cells with hyperactive WNT signaling.

**Figure 8:**
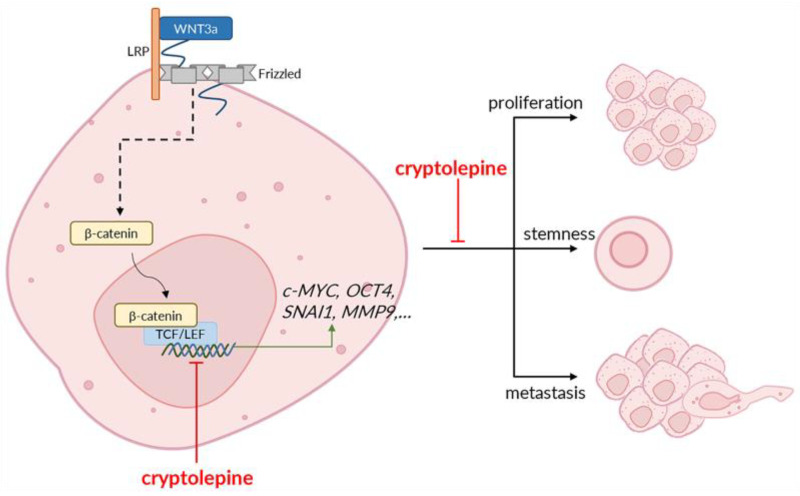
Proposed mechanism through which cryptolepine inhibits CRC progression via WNT/β-catenin signaling. WNT3a interacts with its cognate receptor to trigger a series of intracellular events that culminate in the upregulation of WNT target genes. Cryptolepine downregulates the WNT target genes in CRC cells, resulting in the inhibition of proliferation, stemness, and metastasis.

In conclusion, cryptolepine reduces WNT3a-induced expression of WNT target genes and suppresses the proliferation, stemness, and metastatic processes of CRC cells (Figure 8). Our findings suggest that the effect of cryptolepine on the WNT/β-catenin pathway is specific for CRC cells with hyper-activated WNT signals. Thus, cryptolepine may be best used against WNT-driven (WNT-mutant) cancers. Moreover, while both cell lines used in this study have a mutant *APC* gene, COLO205 has additional *β-catenin* and *BRAF* mutations, whereas DLD1 has an additional *KRAS* mutation [58]. It is uncertain how the additional mutations will affect the activity of cryptolepine. However, the ability of cryptolepine to inhibit the anticancer properties of both cell lines is a favorable outcome as it indicates that in spite of the additional mutations that may cause CRC, cryptolepine may be an effective therapeutic option. Further in vivo studies are required to confirm our findings. Novel evidence may, in the future, confirm the administration of cryptolepine to be an effective treatment for colorectal cancer patients.

## 4. Materials and Methods

### 4.1. Cell Lines and Culture

The COLO205 and DLD1 cell lines used in this study were originally obtained from the American Type Culture Collection and were a kind gift from Professor Regina Appiah-Opong of the Department of Clinical Pathology at the Noguchi Memorial Institute for Medical Research, Ghana. The cells were maintained in Roswell Park Memorial Institute (RPMI) 1640 supplemented with 10% fetal bovine serum (FBS) and 1% penicillin-streptomycin-glutamine (all purchased from Gibco-life technologies, Carlsbad, CA, USA), at 37 °C in a humidified atmosphere containing 5% CO_2_.

### 4.2. Compounds/Drugs

Cryptolepine (C7124-10MG), XAV 939 (X3004-5MG), and human WNT3a (H17001-10UG) were purchased from Sigma-Aldrich, St. Louis, MO, USA. XAV 939 is a specific inhibitor of WNT signaling, while human WNT3a is an activator of WNT signaling. The compounds were prepared as instructed by the manufacturer i.e., cryptolepine and XAV 939 were dissolved in dimethyl sulfoxide and WNT3A was reconstituted with 1× phosphate-buffered saline (PBS).

### 4.3. Cell Viability Assay

The effects of cryptolepine, XAV 939, and WNT3a on COLO205 and DLD1 viability were determined using an MTT assay. Briefly, the cells were seeded into 96-well plates at a density of 1 × 10^4^ cells/well and incubated at 37 °C for 24 h. They were treated with cryptolepine or XAV 939 for 24, 48, and 72 h. For WNT3a, treatment was conducted for 24 h. Then 20 μL of 2.5 mg/mL 3-(4,5-dimethylthiazol-2-yl)-2,5-diphenyltetrazolium bromide (MTT) (Sigma-Aldrich, St Louis, MO, USA), was added to each well and incubated at 37 °C for 4 h. Afterward, 100 μL of acidified isopropanol was added to each well and incubated at 37 °C for 30 min. Absorbance was read at 570 nm with a Varioskan™ LUX multimode microplate reader (Thermo Fisher Scientific, Carlsbad, CA, USA). From the absorbance values, percent cell viabilities were calculated.

### 4.4. Colony Formation/Clonogenic Assay

To assess the ability of the compounds to impact colony formation, the cells were seeded into 6-well plates at a density of 2 × 10^3^ cells/well and incubated at 37 °C for 24 h. The cells in the WNT3a, WNT3a-cryptolepine, and WNT3a-XAV 939 groups were treated with 100 ng/mL of WNT3a for 24 h. Following this, the cells were treated or untreated with cryptolepine or XAV 939 for 72 h. At 3 days post-treatment, the culture medium was removed and fresh medium without drugs was added. The medium was changed every 3 days for 14 days. Then, the cells were washed with 1× PBS, fixed with 4% paraformaldehyde for 10 min, and stained with crystal violet dye (0.1% *w*/*v*) for 15 min. Excess dye was washed with 1× PBS and images were taken with an OPTIKA^®^ microscope (OPTIKA, Ponteranica, Italy). *ColonyArea* plugin in ImageJ (NIH, Bethesda, MD, USA) was used to determine clonogenic cell growth [59].

### 4.5. Wound Healing Assay

A wound healing assay was performed to determine the effect of the compounds on cell migration. Briefly, 70 μL of cell suspension containing 2 × 10^4^ cells were seeded into each chamber of a 35 mm μ-dish with culture-insert (Ibidi GmbH, Munich, Germany) placed in a 6-well plate, and incubated at 37 °C for 24 h. The cells in the WNT3a, WNT3a-cryptolepine, and WNT3a-XAV 939 groups were treated with 100 ng/mL of WNT3a for 24 h. Then, the inserts were removed and the cells were treated or untreated with cryptolepine or XAV 939 for 48 h. Cell migration was monitored with an OPTIKA^®^ microscope (OPTIKA, Ponteranica, Italy) at 0, 24, and 48 h. The images were analyzed with the *Wound Healing Size Tool* plugin in ImageJ (NIH, Bethesda, MD, USA) to determine the percentage closure of the wound [60].

### 4.6. Reverse-Transcription Quantitative PCR (RT-qPCR)

COLO205 and DLD1 cells were seeded into 6-well plates at a density of 1 × 10^6^ cells/well and treated as described. Total RNA was extracted using a Quick-RNA™ MiniPrep Plus Kit (Cat. No. R1057: Zymo Research, Irvine, CA, USA) per the manufacturer’s protocol. Using a Luna Universal One-Step RT-qPCR kit (New England Biolabs, Ipswich, MA, USA), RT-qPCR was performed to determine the mRNA expression of *β-catenin*, *c-MYC*, *WISP1*, *CD133*, *OCT4*, *SNAIL1*, *TWIST1*, *MMP2*, and *MMP9* genes. A QuantStudio™ 5 RT-PCR System (Thermo Fisher Scientific, Carlsbad, CA, USA) was used for reverse transcription and amplification. Thermocycling conditions were reverse transcription (55 °C for 15 min), initial denaturation (95 °C for 1 min), 40 cycles of denaturation (95 °C for 15 s), annealing (different temperatures depending on target gene for 15 s), and extension (60 °C for 1 min). The housekeeping gene *GAPDH* was used as an internal control. Sequences and annealing temperatures of primers are listed in Supplementary Table S1. QuantStudio™ Design & Analysis Software (Life Technologies, Carlsbad, CA, USA) was used to obtain CT values, and the 2^−ΔΔCT^ method [61] was used to determine the relative expression of each gene.

### 4.7. Statistical Analyses

Data were analyzed using GraphPad Prism 9.1.2 (GraphPad Software, San Diego, CA, USA). One-way analysis of variance (ANOVA) followed by Dunnett’s post hoc test was used to compare differences between multiple groups. Data are presented as the mean ± standard error of the mean (SEM) of at least three independent experiments performed in triplicate. Group differences were considered statistically significant when *p* ≤ 0.05.

## Figures and Tables

**Table 1:** IC50 values for treatment conditions.

Cell Lines	Time (h)	IC50 (µM) of Compounds	
		Cryptolepine	XAV 939
**COLO205**	24	5.01 ± 0.43	32.15 ± 3.19
	48	2.45 ± 0.59	24.87 ± 2.45
	72	1.43 ± 0.15	16.51 ± 0.23
**DLD1**	24	3.51 ± 0.31	36.51 ± 1.04
	48	1.16 ± 0.07	25.59 ± 3.22
	72	0.86 ± 0.02	19.19 ± 2.70

## Data Availability

The data presented in this study are available in the article and supplementary material.

## Supplementary Materials

The following supporting information can be downloaded at: https://www.mdpi.com/article/10.3390/ph16071026/s1, Table S1: Sequences and annealing temperatures of primers.

## Abbreviations

CRC: colorectal cancer, CSC: cancer stem cell, ECM: extracellular matrix, EMT: epithelial-mesenchymal transition; FZD: frizzled, ISC: intestinal stem cell, MMP: matrix metalloproteinase, OCT4: octamer-binding transcription factor 4, TCF/LEF: T cell factor/lymphoid enhancer factor, TGF-β: transforming growth factor-β, WISP1: WNT-induced secreted protein 1, WNT: wingless integrated type-1.
