# Supplementary material for: Cryptolepine Suppresses Colorectal Cancer Cell Proliferation, Stemness, and Metastatic Processes by Inhibiting WNT/β-Catenin Signaling
Source: Pharmaceuticals (Basel). 2023 Jul 19;16(7):1026. doi: 10.3390/ph16071026 (PMC10383422; doi:10.3390/ph16071026)
Supplement: Supplementary file 1 [file pharmaceuticals-16-01026-s001.zip › pharmaceuticals-2454534-supplementary.pdf]

Supplementary Material

Cryptolepine Suppresses Colorectal Cancer Cell Proliferation, Stemness and Metastatic Processes by Inhibiting WNT/β-Catenin Signaling

Jude Tetteh Quarshie, Kwadwo Fosu, Nicholas Awuku Offei, Augustine Kojo Sobo, Osbourne Quaye and Anastasia Rosebud Aikins *

West African Centre for Cell Biology of Infectious Pathogens (WACCBIP), Department of Biochemistry Cell and Molecular Biology, University of Ghana, Accra P.O. Box LG 54, Ghana; judequarshie.pub@gmail.com (J.T.Q.); kfosu004@st.ug.edu.gh (K.F.); offeinicholas23@gmail.com (N.A.O.); aksobo@st.ug.edu.gh (A.K.S.); oquaye@ug.edu.gh (O.Q.)

* Correspondence: araikins@ug.edu.gh

Table S1: Sequences and annealing temperatures of primers.

Gene	Primer Sequences		Annealing temperature (°C)
	Forward	Reverse	
GAPDH	GGAGCGAGATCCCTCCAAAAT	GGCTGTTGTCATACTTCTCATGG	56
CTNNB1	CTTCACCTGACAGATCCAAGTC	CCTTCCATCCCTTCCTGTTTAG	58
WISP1	ACTCATTAAGGCAGGGAAGAAG	CCACAGTACTTGGGTTGATAGG	58
c-MYC	AAGCTGAGGCACACAAAGA	GCTTGGACAGGTTAGGAGTAAA	58
OCT4	GTGGAGGAAGCTGACAACAA	CAGGTTTTCTTTCCCTAGCT	56
CD133	ACATGAAAAGACCTGGGGG	GATCTGGTGTCCCAGCATG	55
TWIST1	AGGCATCACTATGGACTTTCTC	GGCCAGTTTGATCCCAGTAT	58
SNAI1	CCACGAGGTGTGACTAACTATG	ACCAAACAGGAGGCTGAAATA	56
MMP2	CTCATCGCAGATGCCTGGAA	TTCAGGTAATAGGCACCCTTGAAGA	60
MMP9	ACGCACGACGTCTTCCAGTA	CCACCTGGTTCAACTCACTCC	60
